# Quantitative assessment of the diagnostic role of FHIT promoter methylation in non-small cell lung cancer

**DOI:** 10.18632/oncotarget.14256

**Published:** 2016-12-27

**Authors:** Xin Geng, Weilin Pu, Yulong Tan, Zhouyi Lu, An Wang, Lixing Tan, Sidi Chen, Shicheng Guo, Jiucun Wang, Xiaofeng Chen

**Affiliations:** ^1^ Department of Cardiothoracic Surgery, Huashan Hospital, Fudan University, Shanghai 200032, China; ^2^ State Key Laboratory of Genetic Engineering, Collaborative Innovation Center for Genetics and Development, School of Life Sciences, Fudan University, Shanghai 200433, China; ^3^ Department of Chest Surgery, Shanghai Pulmonary Hospital, Shanghai 200433, China; ^4^ Department of Bioengineering, University of California at San Diego, La Jolla, CA 92093, USA

**Keywords:** FHIT, DNA methylation, non-small cell lung cancer, NSCLC, diagnosis

## Abstract

Aberrant methylation of CpG islands acquired in promoter regions plays an important role in carcinogenesis. Accumulated evidence demonstrates *FHIT* gene promoter hyper-methylation is involved in non-small cell lung cancer (NSCLC). To test the diagnostic ability of *FHIT* methylation status on NSCLC, thirteen studies, including 2,119 samples were included in our meta-analysis. Simultaneously, four independent DNA methylation datasets from TCGA and GEO database were analyzed for validation. The pooled odds ratio of *FHIT* promoter methylation in cancer samples was 3.43 (95% CI: 1.85 to 6.36) compared with that in controls. In subgroup analysis, significant difference of *FHIT* gene promoter methylation status in NSCLC and controls was found in Asians but not in Caucasian population. In validation stage, 950 Caucasian samples, including 126 paired samples from TCGA, 568 cancer tissues and 256 normal controls from GEO database were analyzed, and all 8 CpG sites near the promoter region of *FHIT* gene were not significantly differentially methylated. Thus the diagnostic role of *FHIT* gene in the lung cancer may be relatively limited in the Caucasian population but useful in the Asians.

## INTRODUCTION

Lung cancer is a complicated disease involving genetic and epigenetic variation, and is one of the leading causes of cancer death all over the world [1]. Lung cancer is often lacking of symptoms in its early stages, however, the five–year survival rate can be increased from 5% to 63% with the early stage of NSCLC thus showing the importance of early diagnosis of NSCLC [2, 3]. DNA methylation is one of the epigenetic modifications in eukaryote, which regulates genes and microRNAs expression [4] and alternative splicing events [5]. It has been observed and confirmed that DNA methylation change is wide-spread in tumor tissues. Hence, with the advantages like good chemical stability, non-invasive detection ability, quantitative signal, reasonable cost and low requirements for sample quality [6], DNA methylation could be a promising biomarker in early cancer detection.

*FHIT* (fragile histidine triad) belongs to the histidine triad gene family, which encodes Hydrolase of Ap3A [7], and the *FHIT*-Ap3A enzyme-substrate complex appears to be the tumor suppressor signal [8]. *FHIT* is located on chromosome 3 and encompasses the common fragile site FRA3B. As a result, translocations and aberrant transcripts of *FHIT* are frequently occurred by carcinogen-induced damages [9]. *FHIT* loss was observed in 64% of non-small-cell lung cancer patients and was significantly associated with squamous cell carcinoma and poor tumor grade [10]. In addition, aberrant transcripts of *FHIT* have been found in other kinds of tumors, such as gastric [11], esophageal [12], and colon carcinomas [13]. *FHIT* has been recently seen as a genome caretaker which is of great importance for genome stability. Multiple studies have found the reduction of *FHIT* expression in precancerous lesions, indicating its potential suppressing role in carcinogenesis [14–19]. The *FHIT* −/− mice were more prone to develop carcinogen-induced tumors as well as the spontaneous tumors than wild type mice [20, 21]. And *FHIT* viral gene therapy was found to be able to prevent and reverse carcinogen-induced tumors in a gastric cancer mouse model [22]. Moreover, recent studies have found that *FHIT* can also function as the tumor suppressor by inhibiting EMT [23, 24]. In summary, *FHIT* is now considered as a cancer suppressor gene and the loss or aberrant transcripts of *FHIT* may be associated with carcinogenesis.

In this study, we performed a meta-analysis to evaluate the ability to use *FHIT* methylation level for early lung cancer diagnosis. Moreover, we searched The Cancer Genome Atlas project (TCGA) as well as the Gene Expression Omnibus (GEO) database, collecting hundreds of NSCLC samples with whole genome DNA methylation datasets and comprehensive clinical information to validate our meta-analysis and correct for the publication bias [25]. Several studies have showed the improved robustness of combining data from papers and databases [26, 27]. Therefore, we innovatively integrated the high-throughput data and published articles to assess and validate the diagnostic ability of *FHIT* methylation test in NSCLC.

## RESULTS

### Study characteristics

**Figure 1:**
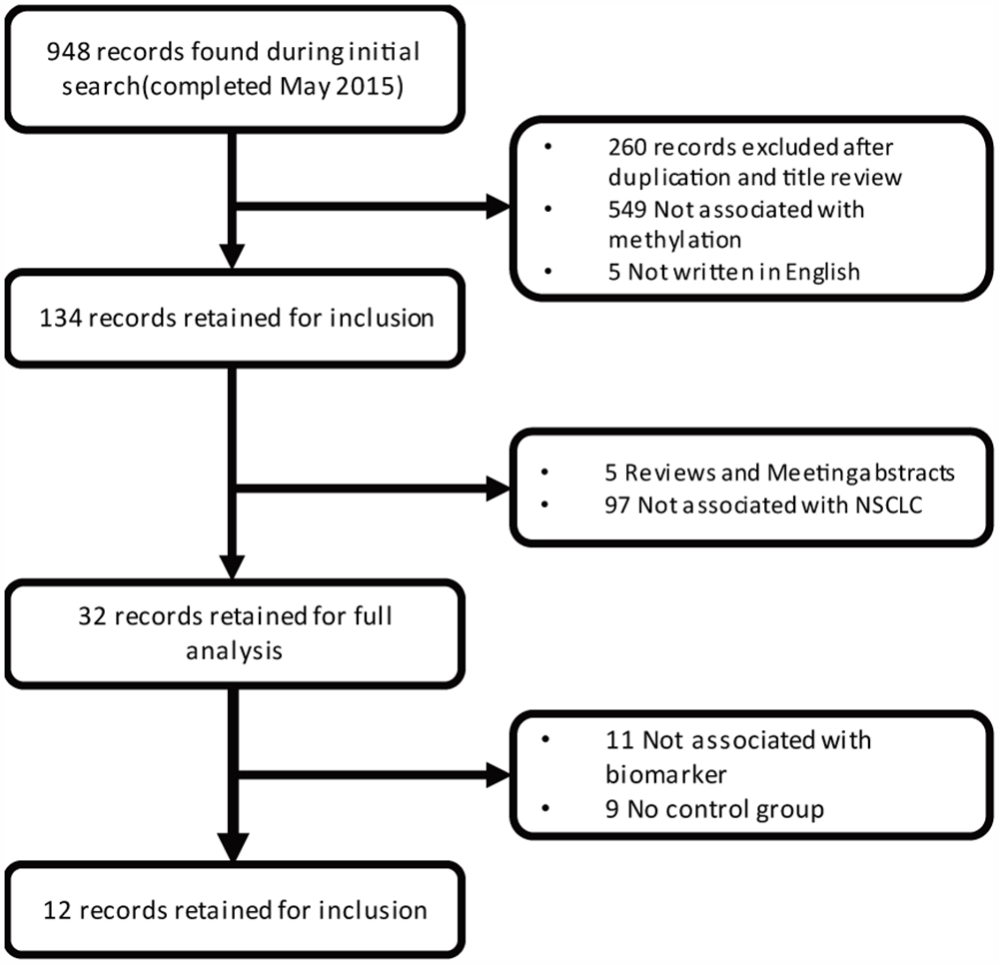
Flow chart of the literature collection procedure

**Figure 2:**
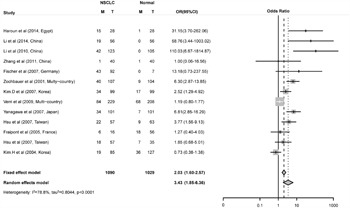
Combined estimates for the association between FHIT promoter hyper-methylation and non-small cell lung cancer (NSCLC) with forest plot Author, year, country of the studies and methylated (M) and total number of the sample (T) in case and control, combined odds ratio (OR) with 95% confidence region were labeled in the left column of the figure. The DerSimonian-Laird estimator and Mantel-Haenszel method were selected to conduct combination estimation for the random effects model and fixed effect model, respectively.

Based on our search strategy, we firstly identified 948 potentially relevant articles (Medline, 229; Web of science, 549; Embase, 170; Cochrane Library, 0). Reference lists including reviews from the relevant articles were also manually screened for inclusion. More detailed information about the inclusion or exclusion criteria was shown in Figure 1. Finally, 12 studies [28–39] were pooled for analysis (Figure 2 and Table 1). The selection of the criteria was described in method section. All these articles were written in English. In total, 1090 lung cancer tissues/plasma and 1029 normal counterpart tissues/plasma were collected. The age of the subjects in the 12 studies ranged from 28 to 86, with mean or median age ranging from 53 to 68. The proportions of stage I samples in the 12 studies differed from 0 to 67.33%, and the percentage of male individuals in the NSCLC samples has a range of 65.2 to 83.8% (Table 1). As for the study aim, 4 articles were especially aiming at NSCLC diagnosis, while the others were designed for the NSCLC prognosis or pathogenesis. For the methylation status detection methods, 10 of the 12 inclusions were conducted with methylation-specific polymerase chain reaction (MSP), while others performed quantitative MSP (Methylight). In addition, three kinds of methylation detection primers were designed for most of the 12 studies (Supplementary Table 1).

**Table 1:** Characteristics of eligible studies considered in the report

Author	Sample Type	Age^a^	Stage I%	Stage (I+II) %	Gender Ratio	Patients (M/T)	Control (M/T)	Method	Aim	Multiple Target	Control design	Ad/Sc	Primer set
Haroun et al	Tissue	53.00	0.18	0.57	0.71	15/28	1/28	qMSP	Non-Diagnosis	Multi	Homogeneity	1.78	1
Li et al	Serum	53.15	0.27	0.39	NA	19/56	0/56	MSP	Non-Diagnosis	Multi	Heterogeneity	0.59	1
Li et al	Serum	55.03	NA	NA	0.71	42/123	0/105	MSP	Non-Diagnosis	Single	Heterogeneity	0.61	1
Zhang et al	Tissue	59.00	0.32	0.74	0.74	1/40	1/40	MSP	Diagnosis	Multi	Homogeneity	0.84	2
Fischer et al	Serum	60.90	0.00	0.00	0.65	43/92	0/7	MSP	Non-Diagnosis	Multi	Heterogeneity	1.71	1
Zochbauer et al	Tissue	61.00	0.57	0.77	0.71	40/107	9/104	MSP	Non-Diagnosis	Single	Homogeneity	1.05	1
Kim.D et al	Tissue	63.00	0.57	0.75	0.81	34/99	17/99	MSP	Non-Diagnosis	Multi	Homogeneity	0.62	3
Verri et al	Tissue	63.90	0.65	NA	0.84	84/229	68/208	MSP	Non-Diagnosis	Single	Homogeneity	1.11	1
Yanagawa et al	Tissue	68.10	0.67	0.74	0.71	34/101	7/101	MSP	Non-Diagnosis	Multi	Homogeneity	1.59	1
Hsu et al^b^	Tissue	69.00	NA	0.65	0.71	22/57	9/63	qMSP	Diagnosis	Multi	Homogeneity	0.76	2
Fraipont et al	Serum	NA	NA	NA	NA	6/16	18/56	MSP	Diagnosis	Multi	Heterogeneity	NA	1
Hsu et al^b^	Serum	NA	NA	0.65	0.71	18/57	7/35	qMSP	Diagnosis	Multi	Heterogeneity	0.76	2
Kim.H et al	Serum	NA	0.59	1.00	0.67	19/85	36/127	MSP	Diagnosis	Multi	Heterogeneity	0.72	1

a mean or median age from articles;

b with two records since there are Tissue and serum data simultaneously in this article. M and T means methylation positive and total, respectively.

### Meta-analysis and heterogeneity source identification

**Figure 3:**
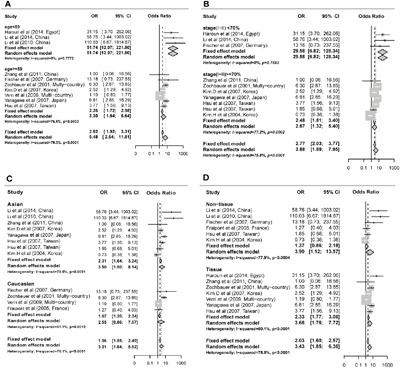
Subgroup meta-analysis for the relationship between FHIT promoter hypermethylation and non-small cell lung cancer (NSCLC) **A**. Subgroup meta-analysis based on age. **B**. Subgroup meta-analysis based on stage(I+II) %. **C**. Subgroup meta-analysis based on race. **D**. Subgroup meta-analysis based on sample type.

The odd ratio (OR) for *FHIT* methylation in cancer group was 3.43 (95% CI: 1.85 - 6.36) in random effects model, and 2.03 (95% CI: 1.60 - 2.57) in fixed effects model, indicating a slight increase of methylation in lung cancer tissues (Figure 2). Comprehensive subgroup analyses were also conducted based on different subtypes, lincluding sample types (tissue or plasma), age, counterpart categories (autogenous or heterogeneous), proportion of stage I, proportion of stage I and II, proportion of male, aim of the study (diagnosis or non-diagnosis), ratio of adenocarcinoma to squamous (Ad/Sc) and other potential confounding factors (Supplementary Table 2). Significant differences were found between the ORs of the younger (51.4, 95% CI: 12.07 - 221.80) and older (3.30, 95% CI: 1.64 - 6.64) subgroup (Figure 3A) and between the ORs of higher (29.58, 95% CI: 6.82 - 128.37) and lower (2.67, 95% CI: 1.32 - 5.40) proportion of stage I and II subgroup (Figure 3B). Interestingly, difference was found between Asian (3.50, 95% CI: 1.50 - 8.14, P = 0.005) and Caucasian population (2.55, 95% CI: 0.86 - 7.57, P = 0.09) subgroup (Figure 3C), and the differential methylation in Caucasian population is not significant, indicating that diagnostic ability of *FHIT* methylation might be limited in Caucasian population. Both tissue and plasma groups showed significant association between *FHIT* methylation and NSCLC (OR = 3.68 and 3.89, respectively) (Figure 3D), which suggested that *FHIT* methylation test is a promising biomarker for NSCLC diagnosis with either tissue or plasma samples. *FHIT* has been reported to be related with smoking history but not with cancer, thus we conducted the subgroups of the percentage of smoking samples. And we found no significant difference between the smoker%<68% and smoker% >=68% subgroups (Supplementary Figure 7). In addition, significant difference between cases and controls was found in both subgroups of MSP and qMSP (OR = 3.22 and 4.31, respectively), suggesting the robustness of both methods in detecting the methylation status of *FHIT* promoter region. Heterogeneity analysis revealed that heterogeneity existed among 13 studies (I^2^ = 78.8%, Q^2^ = 61.05, P < 0.0001) (Figure 2), whereas age, aim and stage were significant heterogeneity resources. The trend in ORs was inversely correlated with age (beta = -3.92, P = 0.05), and age counted for 40.03% of total variance. The aim and stage were also two important heterogeneity sources (P = 0.028 and 0.006), explaining about 51.44% and 17.07% of overall heterogeneity respectively. Other factors such as sample type, proportion of males, detection methods, failed to explain the heterogeneity (Table 2).

**Table 2:** Meta-regression analysis for the main potential interference factors with random-effects model

Subgroup	Coefficient (95% CI)	P-value	τ^2^	QE	QE.P-value
Sample Type	0.18 (-1.14, 1.49)	0.793	0.90	52.84	1.92×10^-7^
Age	-0.15 (-0.3, 0.00)	0.052	0.94	40.04	3.16×10^-6^
Stage I	-3.92 (-7.89, 0.05)	0.052	0.76	35.74	8.13×10^-6^
Stage (I+II)	-3.98 (-6.83, -1.14)	0.006	0.32	17.07	0.02937
Gender Ratio	-5.38 (-18.19, 7.44)	0.411	1.03	46.81	4.26×10^-7^
Methods	0.33 (-1.14, 1.81)	0.656	0.85	53.51	1.45×10^-7^
Aim	1.45 (0.15, 2.74)	0.028	0.88	51.44	3.44×10^-7^
Multiple Target	0.36 (-1.23, 1.95)	0.655	1.08	55.52	6.22×10^-8^
Control Design	0.18 (-1.14, 1.49)	0.793	0.90	52.84	1.92×10^-7^
Ad/Sc	1.04 (-0.74, 2.82)	0.251	0.92	51.41	1.47×10^-7^
Race	-0.30(-1.70, 1.10)	0.673	0.93	48.40	5.25×10^-7^

Bold P-values lower than 0.05 indicate the item would be a significant heterogeneity. QE is used to test for residual heterogeneity in meta regression analysis.

In order to give a robust estimation and bias analysis of our results, a funnel plot of was conducted and the result showed a significant publication bias (Egger test, z =2.76, P = 0.019) and 7 studies exceeded the 95% confidence intervals (Supplementary Figure 1). The adjusted pooled OR after the trim and fill analysis was 2.09 (95% CI: 1.10 - 3.96, P = 0.024) in the random effects model indicating a significantly positive association between *FHIT* methylation and NSCLC (Supplementary Figure 2). Moreover, sensitivity analysis was also applied, the overall ORs were between 2.97 (95% CI: 1.64 - 5.37) and 4.10 (95% CI: 2.17 - 7.76) in the random effects method, indicating the combined OR was consistent and reliable (Supplementary Figure 3). Finally, the cumulative meta-analysis at the time of the published literature found the OR was tending to be stable (Supplementary Figure 4).

### Validation with independent TCGA and GEO lung cancer datasets

**Figure 4:**
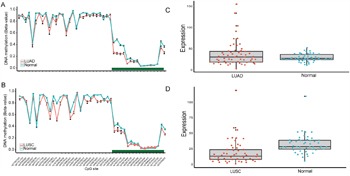
CpG sites on the HM450K Beadchip across FHIT gene region and Gene expression scatterplot with paired data from TCGA dataset Methylation and gene expression status for *FHIT* gene (TCGA lung cancer dataset). **A-B**. each represents the different methylation status of lung cancer subtypes versus normal lung tissues in different datasets. For A-B, the x-axis shows the different CpG sites in *FHIT* genes and the y-axis shows the beta value of each CpG site to represent the methylation level of each CpG site. The green regions in A-B represents the CpG island region of *FHIT*. **C-D**. represents the gene expression status of paired samples. The x-axis of the two figures shows the different types and y-axis shows the gene expression level using RPKM as measurement.

In order to validate the above meta-analysis results with independent datasets, we searched and obtained several datasets from TCGA and GEO. For datasets from TCGA, we downloaded lung adenocarcinoma (LUAD) and lung squamous cell carcinoma (LUSC) methylation datasets. Eight CpG sites located in the same CpG islands as the three sets of primers (Table 3) were obtained after data filtering. In LUAD dataset, though five out of the eight CpG sites showed p-values with statistical significant both in Wilcoxon rank sum test and logistic regression, the absolute mean difference was < 0.1 for all (Table 3). As a result, none of the eight CpG sites could be considered as differentially methylated between lung adenocarcinoma tissues and adjacent normal tissues. Concordantly, in the LUSC dataset, 3 out of 8 CpG sites showed a p-value <0.05 after multiple correction but the absolute mean difference of the 3 CpG sites were < 0.1, which was the same as in the LUAD dataset and couldn't be regarded as significant methylated as well (Table 3 and Figure 4).

**Table 3:** Differential *FHIT* methylation, odds ratio between adenocarcinoma, squamous cell carcinoma and their counterparts from TCGA dataset

Type	CpG site	McaM	McoM	Δβ	P-value^a^	P-value^b^	OR^b^	95%CI^b^
**LUAD**	cg22215728	0.12	0.16	-0.04	0.0008	0.0097	4.33	1.93-12.6
	cg15931943	0.10	0.12	-0.02	0.0071	0.1887	1.80	0.91-4.62
	cg02854288	0.11	0.13	-0.02	0.0016	0.0488	2.81	1.29-7.97
	cg19049316	0.03	0.03	-0.00	0.2251	0.1441	1.73	0.93-3.58
	cg26322434	0.03	0.04	-0.01	0.2993	0.1703	1.63	0.89-3.28
	cg24796403	0.04	0.04	-0.00	0.6626	0.4023	1.35	0.73-2.89
	cg16986494	0.04	0.05	-0.01	0.0193	0.0488	3.12	1.33-9.44
	cg12030002	0.04	0.05	-0.01	0.2050	0.2463	1.49	0.82-2.90
**LUSC**	cg22215728	0.10	0.14	-0.04	1.0×10^-5^	0.0006	3.92	2.07-8.44
	cg15931943	0.09	0.10	-0.01	1.0×10^-5^	0.0116	2.51	1.35-5.13
	cg02854288	0.08	0.10	-0.02	1.0×10^-5^	0.0048	2.76	1.50-5.58
	cg19049316	0.03	0.03	-0.00	0.5233	0.3103	0.66	0.26-1.16
	cg26322434	0.03	0.02	-0.01	0.5165	0.2971	0.77	0.45-1.21
	cg24796403	0.04	0.03	-0.01	0.5233	0.2948	0.31	0.03-1.13
	cg16986494	0.04	0.03	-0.01	0.0245	0.5445	0.84	0.26-1.37
	cg12030002	0.04	0.03	-0.01	0.1117	0.1253	0.10	0.005-0.99

McaM and McoM represent the mean of case methylation (Beta) and mean of control methylation (Beta). Methylation levels are calculated with formula: Beta = (M/M + U). P-values^a^ were calculated from Wilcoxon rank sum test after false discovery rate (FDR adjustment). P-value^b^ and OR^b^ and 95%CI^b^ are from logistic regression analysis with P-value^b^ were also after false discovery rate (FDR adjustment).

Because of the conflicting results came from the meta-analysis and TCGA dataset, we obtained other datasets from the GEO website. The first dataset was the combination of GSE39279 and GSE52401. In GSE39279 dataset, 322 lung adenocarcinoma and 122 lung squamous cell tissues were included. While in GSE39279 dataset, a total of 244 normal lung tissues were included, and both of the datasets used the Illumina HumanMethylation450 Bead Chip for methylation measurement. The two datasets were combined and a total of 444 tumor tissues and 244 normal tissues were included in the subsequent analysis. We performed the same analysis as in TCGA dataset and the result was almost the same. Due to the large number of samples, we found all the p-values of the eight CpG sites were < 0.05 even after multiple test correction (Supplementary Table 3 and Supplementary Figure 5). However, the absolute mean difference of the eight CpG sites were < 0.1 and couldn't be considered as significant methylated CpG sites.

Moreover, we downloaded GSE56044 with 124 NSCLC tissues and 12 adjacent normal tissues for further validation. GSE56044 didn't have clinical information on the subtypes of NSCLC and thus we just utilized NSCLC tissues for subsequent comparison. And the result was unsurprisingly the same as the two datasets mentioned before, showing no significant methylation state of the eight CpG sites (Supplementary Figure 6).

### Gene expression data with TCGA RNA-Seq dataset

DNA methylation played a key factor in regulating gene expression. It may be informative to see if the gene expression of *FHIT* was changed due to the very different results obtained from microarray data and the meta-analysis. We downloaded level 3 RNA-Seq data of LUAD and LUSC from TCGA project. However, after calculating the fold change and p-value with multiple correction, no significantly differential expression was shown both in LUAD (P = 0.58, Fold change = 1.30) and LUSC (P = 5.7×10^-7^, Fold change=1.86) when compared with the adjacent normal tissues. Furthermore, the expression level of *FHIT* is relatively low in LUAD (mean RPKM=37.04) and its adjacent normal tissues (mean RPKM: 28.49) as well as in LUSC (mean RPKM=17.29) and its adjacent normal tissues (mean RPKM=32.18), which implied that the role of *FHIT* gene played in NSCLC carcinogenesis need to be further confirmed (Figure 4).

## DISCUSSIONS

The *FHIT* gene loss was observed in 64% of NSCLC patients and is reported to be significantly associate with squamous cell carcinoma and poor tumor grade. However, the diagnostic ability of the methylation status of the *FHIT* gene in lung cancer still remains unclear. We therefore performed an integrated analysis to give a comprehensive evaluation of the diagnostic ability using *FHIT* promoter methylation level as a biomarker in NSCLC. As expected, a significant association was found between *FHIT* methylation and NSCLC in meta-analysis (OR = 3.43), indicating the existence of a strong association between *FHIT* promoter methylation and lung cancer.

In the validation stage, all the results from three independent datasets showed no significance of differential methylation between NSCLC and normal tissues on account of the small mean methylation difference. It was found that in the dataset from TCGA dataset, none of the eight CpG sites which shared the same CpG island with the primers in the meta-analysis is significantly different methylated. And the result is further confirmed by other two datasets from the GEO database. Furthermore, we downloaded the RNA-Seq data from TCGA project and still no significant differential expression of *FHIT* gene was found both in LUAD and LUSC when compared with adjacent normal tissues. Besides, the expression level of the *FHIT* gene is relatively low in comparison with other functional genes in cancer. We should be noticed that all the independent datasets from TCGA and GEO were based on Caucasian population (Supplementary Table 5-6). The result about Caucasian population from datasets is consistent with the result from meta-analysis, so the relationship between *FHIT* methylation and NSCLC in Caucasian population is robust. In addition, we also detected the methylation status of FHIT promoter in other kinds of cancers using TCGA datasets for further validation, and similar results were obtained and showed limited diagnostic ability (Supplementary Table 4). Besides, we need more micro-assay and RNA-Seq data based on Asian population to distinguish whether the diagnostic role of *FHIT* is specific in the Asians.

In our meta-analysis, we found high rate of heterogeneity between the studies (p < 0.0001). Thus we did further research to explore the influential confounding factors. We found that ages, stages as well as the aims are the sources of heterogeneity (Table 2). However, significant odds ratios between *FHIT* promoter methylation and NSCLC were still retained in most of the subgroups, which is in accordance with the overall meta-analysis results (Supplementary Table 2). Subgroup analysis showed that *FHIT* methylation is significantly relevant to NSCLC in Asians (OR = 3.50, 95% CI: 1.50 - 8.14) but not in Caucasian population (OR = 2.55, 95% CI: 0.86 - 7.57), indicating that aberrant methylation of *FHIT* can be a diagnostic biomarker for NSCLC in Asian population. In the comparative analysis with the other studies, Wu et al found differential methylation of *FHIT* promoter in both Caucasian and Asian populations, which was different with our findings [40]. In addition, the much more significant difference of *FHIT* promoter methylation between NSCLC and normal controls was observed in our meta-analysis and in Wu's as well as in Yan's study [41]. The above consistencies and inconsistencies between the three studies implied the need to test the association between *FHIT* methylation and NSCLC with larger sample sizes and more advanced technology.

There are several limitations in our study. Firstly, the strong heterogeneity of the included studies may decrease the statistical power of our results. Secondly, though we have conducted the trim and fill analysis and sensitivity analysis, the publication bias may still present. Thirdly, we have searched the papers only written in English, while many papers written in other languages were ignored. Due to the previous limitations, we strongly recommend to use more advanced methylation detection methods, like WGBS (whole genome bisulfite sequencing) and RRBS (restricted region bisulfite sequencing), to explore the association between *FHIT* promoter methylation and NSCLC with larger sample sizes.

## MATERIALS AND METHODS

### Search strategy, selection of studies and data extraction

This pooled study involved searching a range of computerized databases, including Embase, Cochrane Library, OVID Medline and Web of Science for articles published in English by October 2015. The study used a subject and text word strategy with (*FHIT* OR AP3Aase OR FRA3B) AND (lung cancer) as the primary search terms. Wildcard character of star, dollar or some other truncations were applied according to the rules of the databases to allow effective article collection.

Two independent reviewers (Geng, Guo) screened the titles and abstracts derived from the literature search to identify relevant studies. The following types of studies were excluded: animal and cell experiments, case reports, reviews or meta-analyses and studies of non-case-control studies or studies with insufficient data or those proving inaccessible after making contact with the authors. The remaining articles were further examined to see if they met the inclusion criteria: 1) the patients had to be diagnosed with NSCLC (Ad and Sc), 2) the studies contained *FHIT* gene promoter methylation data from tissue, blood or plasma, 3) the studies had to be case-control studies which included tissue-tissue, blood-blood or plasma-plasma in case and controls respectively, 4) OR can be calculated or extracted from the text. The reference sections of all retrieved articles were searched to identify further relevant articles. Potentially relevant papers were obtained and the full text articles were screened for inclusion by two independent reviewers (Geng, Guo). Disagreements were resolved by discussion with WP, ZL and AW. Included studies were summarized in data extraction forms. Authors were contacted when relevant data were missing. The name of the first author, year of publication, sample size, age (mean or median), gender proportion (male/female, M2F), the proportion of TNM stage I and II samples (proportion of early stage of NSCLC samples), publication aim (for diagnosis or not), analyzing multiple genes or not (one or more genes detected simultaneously in studies design), control type (autogenous or heterogeneous counterpart) and methylation status of the *FHIT* promoter in human NSCLC and normal or control tissues were extracted (Table 1).

### Meta-analysis and heterogeneity source identification

Data were analyzed and visualized mainly using R Software (R version 3.1.0) including meta, metafor and mada packages [42]. The strength of association was expressed as pooled odds ratio (OR) with corresponding 95% confidence intervals (95% CI). Data were extracted from the original studies and recalculated if necessary. Heterogeneity was tested using the I^2^ statistic with values over 50% and Chi-squared test with P ≤ 0.1 indicating strong heterogeneity between the studies. Tau-squared (τ^2^) was used to determine how much heterogeneity was explained by subgroup differences. The data was pooled using the DerSimonian and Laird random effects model (I^2^ > 50%, P ≤ 0.1) or fixed effects model (I^2^ < 50%) according to heterogeneity statistic I^2^[43]. A two-sided P ≤ 0.05 was set as the threshold of being significant without special annotation. With a lack of heterogeneity among included studies, the pooled odds ratio estimates were calculated using the fixed-effects model [44]. Otherwise, the random-effects model was used [45]. Random effects meta-regression was employed to determine how much of the heterogeneity (between-study variance) was explained by the explanatory variables when the heterogeneity was significant. Nine variables were analyzed in meta-regression, including control types (autogenous and heterogeneous), gender proportion, proportion of TNM stage I and II samples, mean or median age (> 59 or ≤ 59), single or multiple target detection, sample types (plasma or tissue), methylation detection methods (MSP, qMSP), study designs (diagnosis or non-diagnosis) and primer sets. Sensitivity analyses were performed to assess the contribution of single study to the final result with the abandonment of one article each time. Publication bias was analyzed by funnel plot with mixed-effects version of the Egger test [46]. If bias was suspected, the conventional meta-trim method was used to re-estimate the effect size.

### TCGA and GEO datasets extraction and analysis

TCGA DNA methylation datasets which included 23 lung adenocarcinoma and 40 lung squamous cell carcinoma tissues as well as 63 paired adjacent tissues, were collected from TCGA project [ http://cancergenome.nih.gov/] using Illumina HumanMethylation 450K Beadchip [47]. And The GEO datasets including GSE39279, GSE52401 and GSE56044 were downloaded from Gene Expression Omnibus [ http://www.ncbi.nlm.nih.gov/geo/], including a sum of 568 NSCLC tissues and 256 adjacent or normal lung tissues [48–50]. All of the above datasets are using Illumina HumanMethylation450 Bead Chip for methylation measurement. The estimation of methylation for each CG probe was calculated between methylated (M) and unmethylated (U) alleles. Specifically:

$$beta\;=\;\frac{\max(M,0)}{\max(M,0)+\max(U,0)}$$

M and U represent the mean signal intensities for about 30 replicates on the array. The methylation signals of the CpG sites in the datasets previously mentioned were all defined according to the beta value. CpG site would be immediately omitted when it was missing in any one or more samples. CpG sites of *FHIT* gene in TCGA dataset and GEO dataset were not completely the same due to the quality control previously mentioned.

P-value was calculated with Wilcoxon rank sum test. To correct for multiple testing, Benjamini and Hochberg procedure was conducted. For identification of differentially methylated CpG sites, adjusted P-value ≤0.05 and absolute mean difference ≥0.1 was set as the criteria. Besides, logistic regression was also conducted to calculate the OR and p-value for every CpG site with Benjamini and Hochberg multiple comparison correction followed. Data was analyzed and visualized mainly with R software (R 3.1.0) [51][52].

### RNA-Seq data extraction and analysis

RNA-Seq data was downloaded from TCGA Data Portal, including 114 lung adenocarcinoma and 104 lung squamous cell carcinoma and 218 paired adjacent normal lung tissues. Level 3 RNA-Seq data was obtained and per million mapped reads (RPKM) was used for gene expression quantification. We assessed the significance of the differential gene expression by comparing the tumor tissues with paired adjacent normal tissues using Wilcoxon rank sum test and following the Benjamini and Hochberg false discovery rate (FDR) correction [52]. For identification of differentially expression genes, adjusted p-value ≤0.05 and fold change ≥2.0 were set as the criteria. All the data analysis was conducted with open-source R software (version 3.1.0).

## CONCLUSION

The diagnostic role of *FHIT* gene in the lung cancer is relatively limited in the Caucasian population but may be useful in the Asians. However, more datasets and studies with large sample sizes are needed for further confirmation.

## SUPPLEMENTARY MATERIALS FIGURES AND TABLES




