# Supplementary material for: Quantitative assessment of the diagnostic role of FHIT promoter methylation in non-small cell lung cancer
Source: Oncotarget. 2016 Dec 27;8(4):6845–56. doi: 10.18632/oncotarget.14256 (PMC5351674; doi:10.18632/oncotarget.14256)
Supplement: Supplementary file 1 [file oncotarget-08-6845-s001.pdf]

Quantitative assessment of the diagnostic role of *FHIT* promoter methylation in non-small cell lung cancer

SUPPLEMENTARY FIGURES AND TABLES

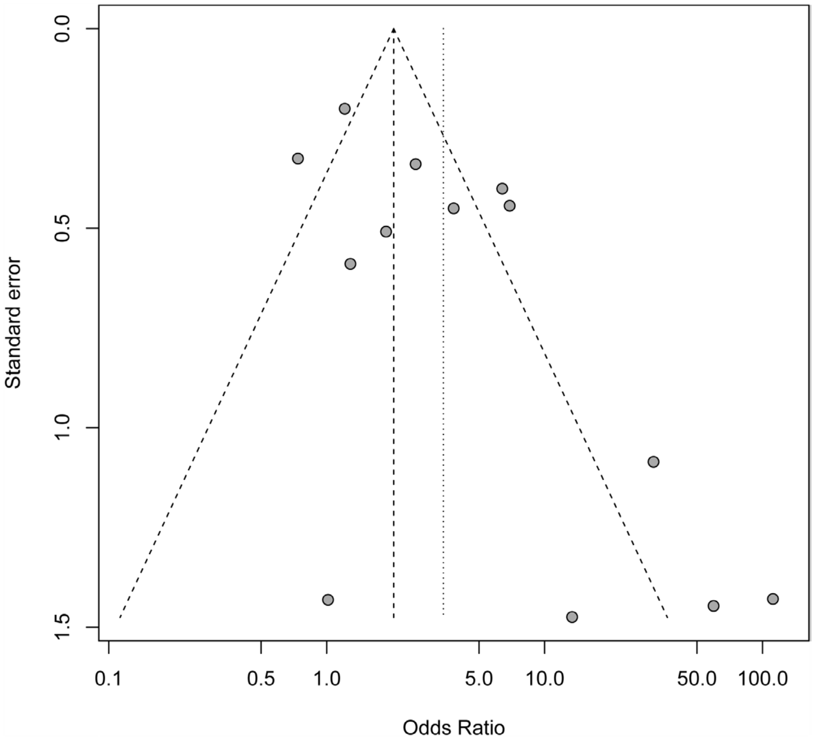

Supplementary Figure 1: Funnel plot to diagnosis of the publication bias.

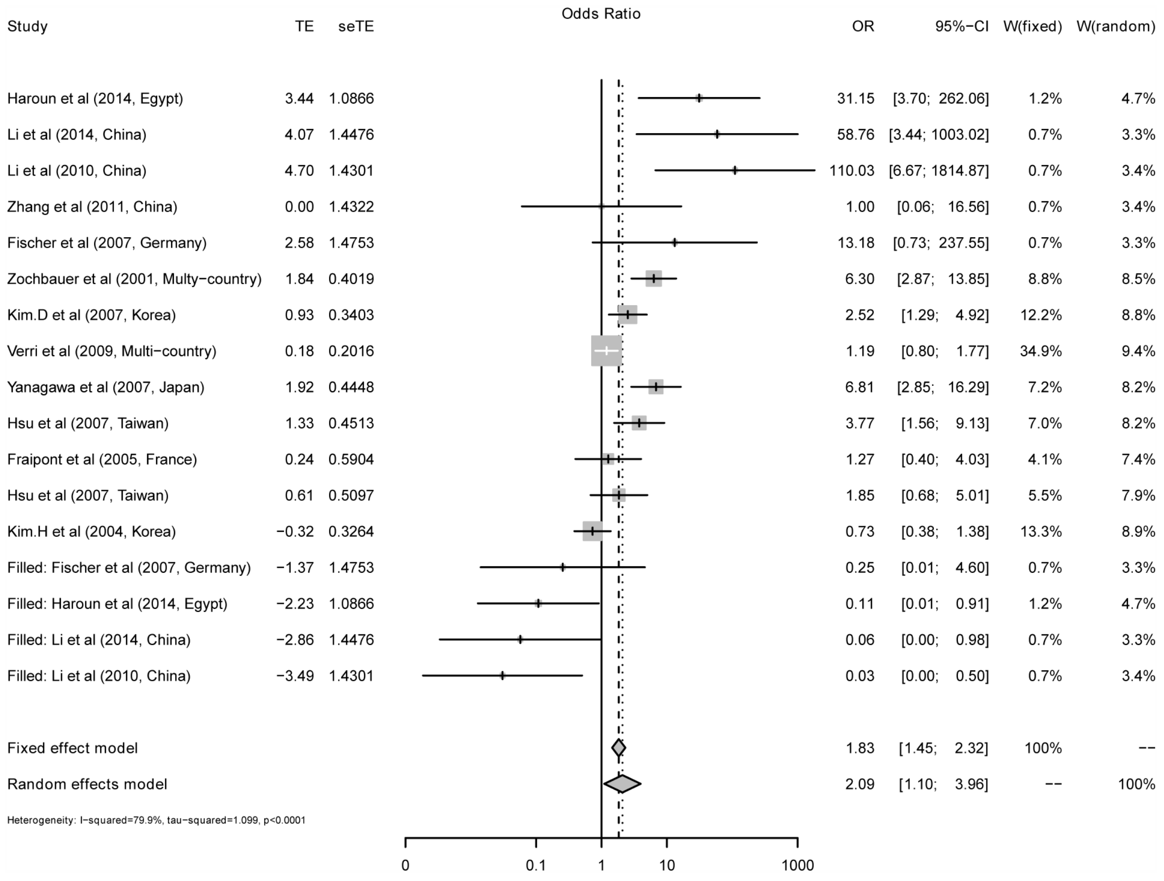

Supplementary Figure 2: Combined estimates for the association between *FHIT* methylation and NSCLC after trim-fill treatment.

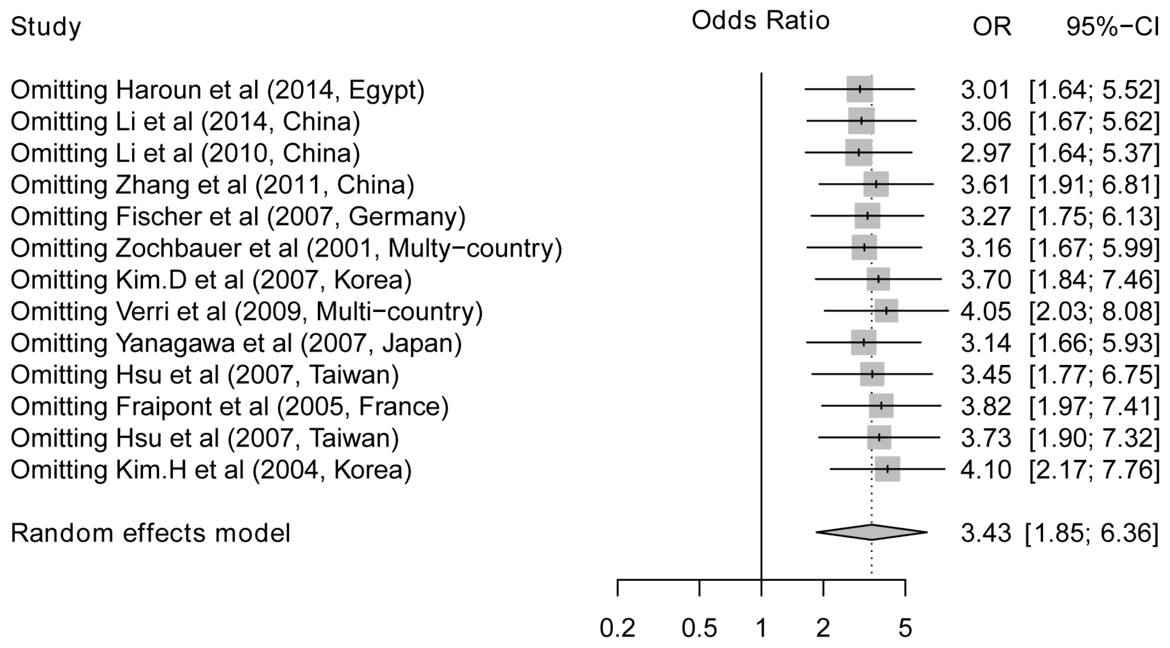

Supplementary Figure 3: Sensitivity analyses of the overall effect by omitting a single study.

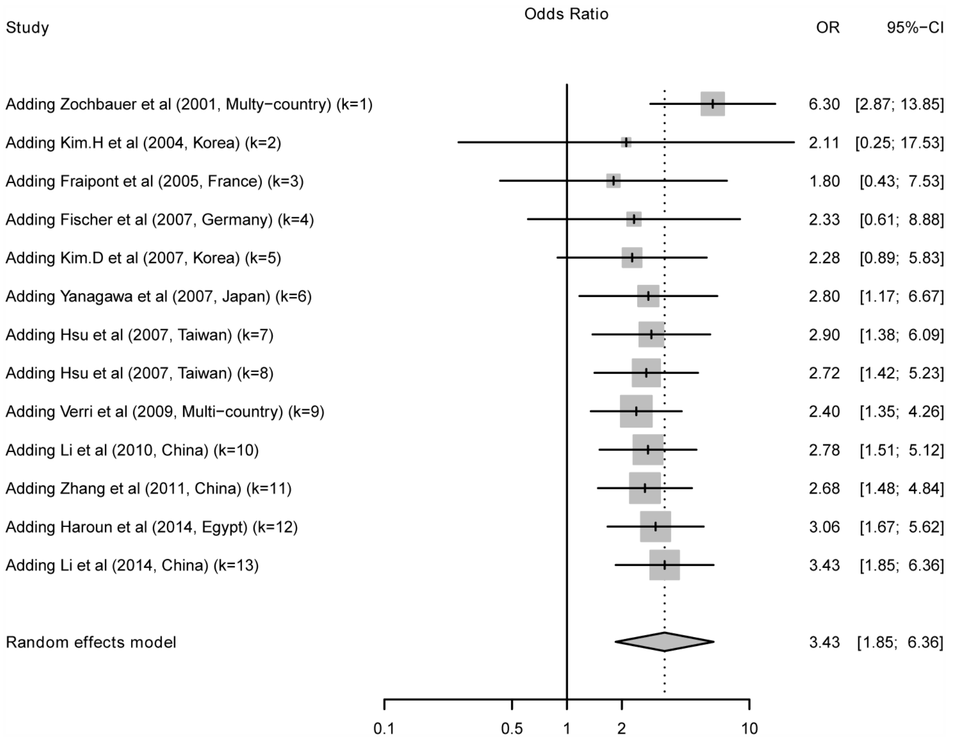

Supplementary Figure 4: Cumulative analysis for the relationship between *FHIT* promoter hypermethylation and non-small cell lung cancer (NSCLC). Cumulative meta-analysis of studies ordered chronologically by publication year with random effects model.

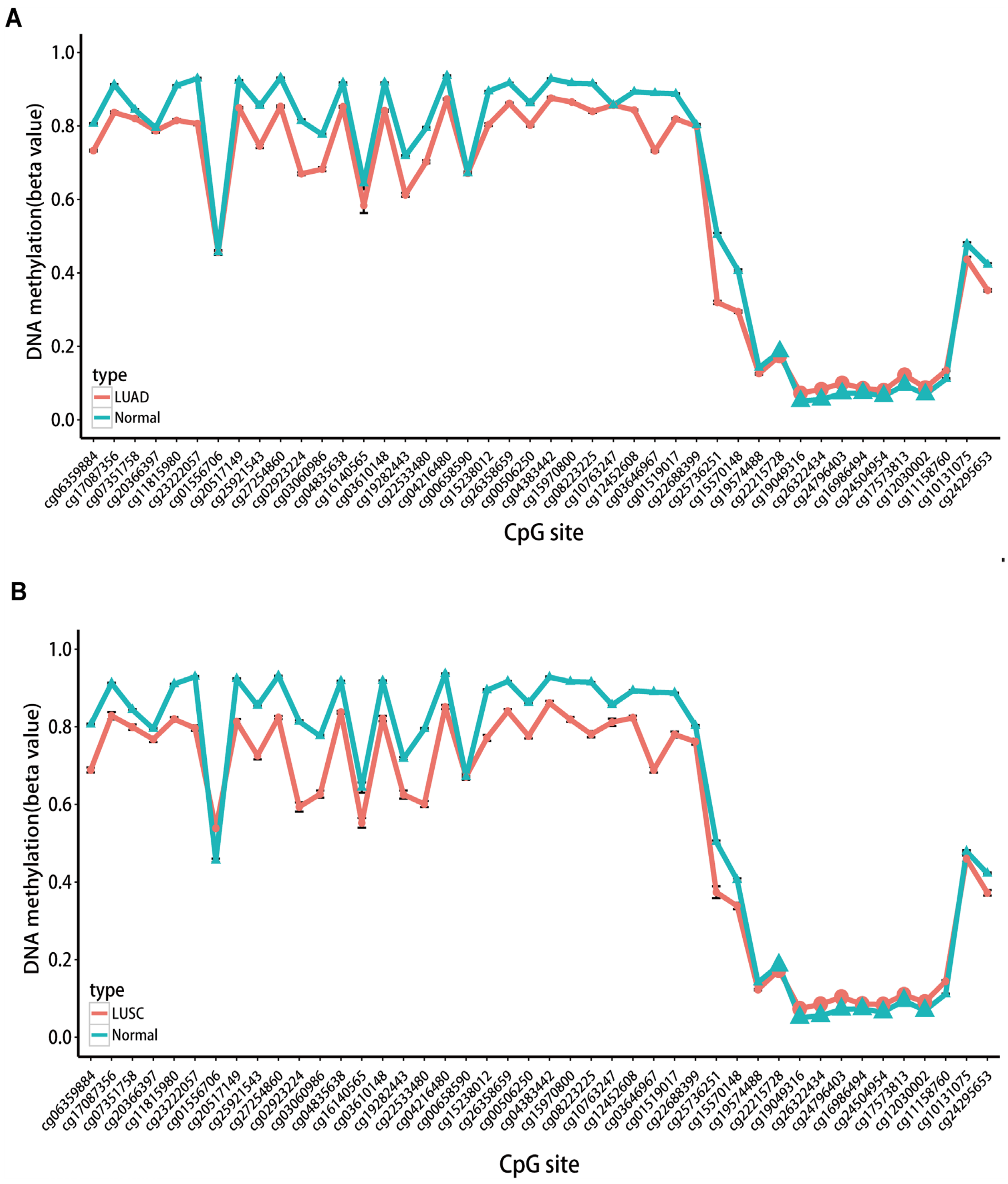

Supplementary Figure 5: CpG sites on the Illumina Infinium HumanMethylation450 Beadchip array across *FHIT* gene region from combining the GSE39279 and GSE52401 datasets. the x-axis shows the different CpG sites in *FHIT* genes and the y-axis shows the beta value of each CpG site to represent the methylation level of each CpG site. **A, B.** represents the methylation status of FHIT promoter region of LUAD and LUSC compared with the normal controls, respectively.

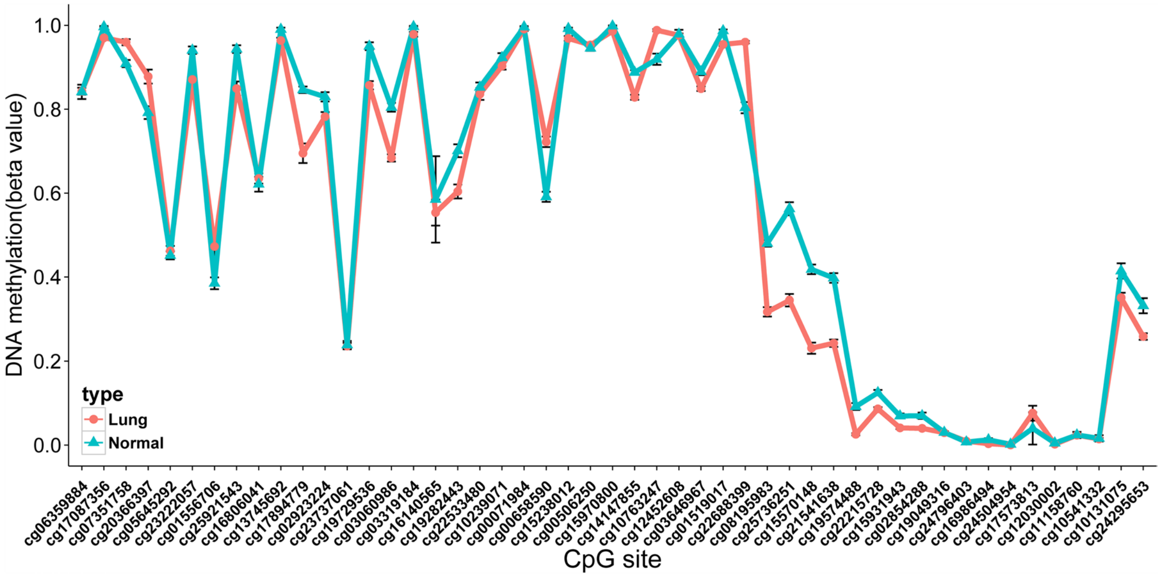

Supplementary Figure 6: CpG sites on the Illumina Infinium HumanMethylation450 Beadchip array across *FHIT* gene region from GSE56044 dataset. the x-axis shows the different CpG sites in *FHIT* genes and the y-axis shows the beta value of each CpG site to represent the methylation level of each CpG site.

Study	OR	95%−CI
**Smoker%<68%**		
Haroun et al (2014, Egypt)	31.15	[3.70; 262.06]
Hsu et al (2007, Taiwan)	3.77	[1.56; 9.13]
Hsu et al (2007, Taiwan)	1.85	[0.68; 5.01]
Li et al (2010, China)	110.03	[6.67; 1814.87]
Zhang et al (2011, China)	1.00	[0.06; 16.56]
**Fixed effect model**	**3.79**	**[2.07; 6.92]**
**Random effects model**	**5.76**	**[1.64; 20.24]**
Heterogeneity: I−squared=67.1%, tau−squared=1.202, p=0.0162		
**Smoker%>=68%**		
Kim.D et al (2007, Korea)	2.52	[1.29; 4.92]
Verri et al (2009, Multi−country)	1.19	[0.80; 1.77]
Yanagawa et al (2007, Japan)	6.81	[2.85; 16.29]
Zochbauer−Muller et al (2001, Multy−country)	6.30	[2.87; 13.85]
**Fixed effect model**	**2.12**	**[1.58; 2.84]**
**Random effects model**	**3.20**	**[1.31; 7.83]**
Heterogeneity: I−squared=86.7%, tau−squared=0.7074, p<0.0001		
**Fixed effect model**	**2.37**	**[1.82; 3.09]**
**Random effects model**	**3.95**	**[1.98; 7.88]**
Heterogeneity: I−squared=78.8%, tau−squared=0.7268, p<0.0001		

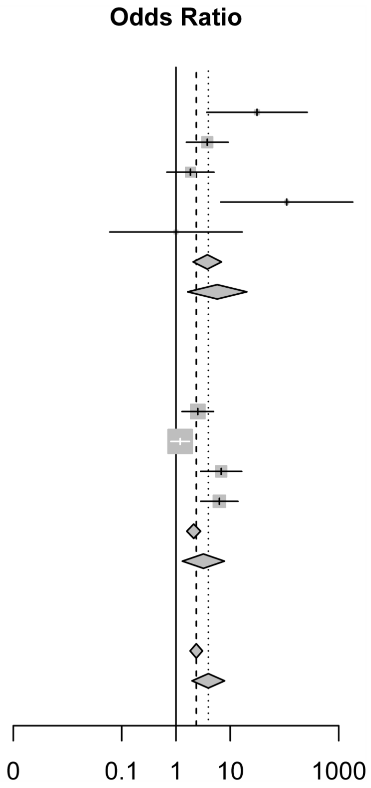

Supplementary Figure 7: Subgroup analysis for the relationship between *FHIT* promoter hypermethylation and non-small cell lung cancer (NSCLC) based on smoking status.

Supplementary Table 1: Three kinds of primers of present 12 studies

Author	Forward	Reverse	CpG island coordinate
Fraipont et al (2005, France)	chr3:61,236,911-61,236,936	chr3:61,236,854-61,236,893	chr3:61236606-61237227
Fischer et al (2007, Germany)	chr3:61,236,911-61,236,936	chr3:61,236,854-61,236,893	chr3:61236606-61237227
Haroun et al (2014, Egypt)	chr3:61,236,911-61,236,936	chr3:61,236,854-61,236,893	chr3:61236606-61237227
Hsu et al (2007, Taiwan)	chr3:61,236,954-61,236,978	chr3:61,236,854-61,236,893	chr3:61236606-61237227
Kim.D et al (2007, Korea)	chr3:61,237,021-61,237,041	chr3:61,236,855-61,236,875	chr3:61236606-61237227
Kim.H et al (2004, Korea)	chr3:61,236,911-61,236,936	chr3:61,236,854-61,236,893	chr3:61236606-61237227
Li et al (2010, China)	chr3:61,236,911-61,236,936	chr3:61,236,854-61,236,893	chr3:61236606-61237227
Li et al (2014, China)	chr3:61,236,911-61,236,936	chr3:61,236,854-61,236,893	chr3:61236606-61237227
Verri et al (2009, Multi-country)	chr3:61,236,911-61,236,936	chr3:61,236,854-61,236,893	chr3:61236606-61237227
Yanagawa et al (2007, Japan)	chr3:61,236,911-61,236,936	chr3:61,236,854-61,236,893	chr3:61236606-61237227
Zhang et al (2011, China)	chr3:61,236,954-61,236,978	chr3:61,236,854-61,236,893	chr3:61236606-61237227
Zochbauer-Muller et al (2001, Multy-country)	chr3:61,236,911-61,236,936	chr3:61,236,854-61,236,893	chr3:61236606-61237227

Supplementary Table 2: Subgroup analysis for the main potential confounding factors with random effects model

Subgroup	Number of study	OR	Lower	Upper	Q	I^2	P-value
Overall	13	3.43	1.85	6.36	61.05	80.00%	<0.00001
Age<59	3	51.7384	12.0688	221.8	0.5	0.00%	
Age>=59	7	3.2956	1.6368	6.6358	25.86	76.80%	0.0008
Stage I<50.45%	4	13.4147	2.4774	72.6401	4.92	39.00%	
Stage I>=50.45%	5	2.3709	1.0634	5.2859	31.5	87.30%	0.0693
Stage I+II<60%	3	29.5812	6.8181	128.3717	0.53	0.00%	
Stage I+II>=60%	7	2.6727	1.3227	5.4005	26.26	77.20%	0.0038
Male%<73.31%	6	4.5011	1.4339	14.1297	33.28	85.00%	
Male%>=71.31%	5	2.6641	1.1865	5.9818	14.84	73.00%	0.4631
Asian	8	3.50	1.50	8.14	32.52	78.00%	
Caucasian	4	2.55	0.86	7.57	15.88	87.00%	0.65
MSP	10	3.2157	1.5466	6.6861	47.87	81.20%	
qMSP	3	4.312	1.3293	13.9871	5.64	64.60%	0.6782
Diagnose	5	1.5016	0.7372	3.0585	9.2	56.50%	
Non-diagnose	8	6.9206	2.7482	17.4273	42.24	83.40%	0.0102
Multiple targets	10	3.1662	1.5526	6.4572	33.37	73.00%	
Single target	3	5.6535	0.9884	32.3359	22.15	91.00%	0.5464
Heterogeneous	6	3.8974	1.1196	13.5668	22.62	77.90%	
Autogenous	7	3.6814	1.7551	7.7222	30.22	80.10%	0.9386
Plasma	6	3.8974	1.1196	13.5668	22.62	77.90%	
Tissue	7	3.6814	1.7551	7.7222	30.22	80.10%	0.9386
Ad2Sc<0.796	6	3.396	1.2897	8.9421	25.32	80.30%	
Ad2Sc>=0.796	6	4.7264	1.5624	14.298	30.26	83.50%	0.6595
Primerset I	9	4.92	1.91	12.69	59.67	87.00%	
Primerset II	3	2.61	1.37	4.98	1.58	0.00%	
Primerset III	1	2.52	1.29	4.92	-	-	0.48

Bold P-values lower than 0.05 indicate significant differences between groups (random effects model, d.f. = 1).

Supplementary Table 3: Differential *FHIT* methylation, odds ratio between adenocarcinoma, squamous cell carcinoma and their counterparts from TCGA dataset

Type	CpG site	Position	McaM	McoM	Δβ	p-value[a]	p-value_lr[b]	OR[b]	95%CI[b]
LUAD	cg22215728	61236652	0.17	0.19	0.01	0.0002	0.005	1.29	1.08-1.54
	cg19049316	61237063	0.07	0.05	0.02	2.83E-69	5.18E-35	0.01	0.003-0.016
	cg26322434	61237156	0.08	0.06	0.02	2.84E-66	1.41E-33	0.006	0.003-0.013
	cg24796403	61237172	0.10	0.07	0.03	3.07E-56	8.62E-32	0.02	0.01-0.04
	cg16986494	61237206	0.09	0.07	0.02	3.33E-15	3.19E-11	0.31	0.22-0.43
	cg24504954	61237217	0.08	0.06	0.02	9.80E-24	2.47E-18	0.16	0.10-0.23
	cg17573813	61237223	0.12	0.09	0.03	2.80E-27	0.026	0.81	0.67-0.97
	cg12030002	61237226	0.09	0.07	0.02	1.62E-34	6.97E-25	0.07	0.04-0.11
LUSC	cg22215728	61236652	0.10	0.14	0.04	1.00E-05	0.00066	3.92	2.08-8.44
	cg15931943	61236909	0.09	0.10	0.01	1.00E-05	0.01163	2.51	1.35-5.13
	cg02854288	61236911	0.08	0.10	0.02	1.00E-05	0.00485	2.76	1.50-5.58
	cg19049316	61237063	0.03	0.03	0.00	0.52339	0.31038	0.67	0.26-1.16
	cg26322434	61237156	0.03	0.02	0.01	0.51658	0.29719	0.77	0.45-1.21
	cg24796403	61237172	0.04	0.03	0.01	0.52339	0.29486	0.31	0.03-1.13
	cg16986494	61237206	0.04	0.03	0.01	0.02458	0.54451	0.83	0.26-1.37
	cg12030002	61237226	0.04	0.03	0.01	0.11177	0.12536	0.10	0.005-0.99

McaM and McoM represent the mean of case methylation (Beta) and mean of control methylation (Beta). Methylation. levels are calculated with formula: Beta = (M/M + U).
Position represents the chromosome position of each CpG site according to GRCh37/hg19.
P-values[a] are calculated from Wilcoxon rank sum test after false discovery rate (FDR adjustment).
P-value[b] and OR[b] and 95%CI[b] are from logistic regression analysis with P-value[b] were also after false discovery rate (FDR adjustment).

Supplementary Table 4: *FHIT* methylation status in other cancer types of TCGA database

See Supplementary File 1

Supplementary Table 5: Population distributions of TCGA LUAD and LUSC datasets

Population	LUAD (n=23)	LUSC (n =40)
Black/African American	4	0
Caucasian	18	33
Unknown	1	6
Asian	0	1

Supplementary Table 6: Population distributions of three GEO datasets

Population	GSE39279 (n=444)	GSE52401(n=244)	GSE56044(n=136)
European	291	0	0
United States	153	0	0
Italian	0	244	0
Swedish	0	0	136
