# Supplementary material for: Quantitative assessment of the diagnostic role of FHIT promoter methylation in non-small cell lung cancer
Source: Oncotarget. 2016 Dec 27;8(4):6845–56. doi: 10.18632/oncotarget.14256 (PMC5351674; doi:10.18632/oncotarget.14256)
Supplement: Supplementary file 2 [file oncotarget-08-6845-s002.docx]

**Supplementary Table 4*. FHIT* methylation status in other cancer types of TCGA database**

| **Cancer Type** | **CpG site** | **Position** | **McaM** | **McoM** | **△β** | **p-value** |
| --- | --- | --- | --- | --- | --- | --- |
| **BRCA** | cg22215728 | 61236652 | 0.10 | 0.09 | 0.01 | 0.41 |
|  | cg15931943 | 61236909 | 0.09 | 0.08 | 0.01 | 0.29 |
|  | cg02854288 | 61236911 | 0.09 | 0.08 | 0.01 | 0.31 |
|  | cg19049316 | 61237063 | 0.03 | 0.03 | 0.00 | 0.32 |
|  | cg26322434 | 61237156 | 0.04 | 0.04 | 0.00 | 0.84 |
|  | cg24796403 | 61237172 | 0.04 | 0.04 | 0.00 | 0.57 |
|  | cg16986494 | 61237206 | 0.04 | 0.04 | 0.00 | 0.62 |
|  | cg12030002 | 61237226 | 0.04 | 0.04 | 0.00 | 0.95 |
| **COAD** | cg22215728 | 61236652 | 0.08 | 0.10 | 0.02 | 0.58 |
|  | cg15931943 | 61236909 | 0.09 | 0.09 | 0.00 | 0.62 |
|  | cg02854288 | 61236911 | 0.09 | 0.09 | 0.00 | 0.81 |
|  | cg19049316 | 61237063 | 0.03 | 0.03 | 0.00 | 0.27 |
|  | cg26322434 | 61237156 | 0.03 | 0.03 | 0.00 | 0.36 |
|  | cg24796403 | 61237172 | 0.04 | 0.04 | 0.00 | 0.98 |
|  | cg16986494 | 61237206 | 0.04 | 0.04 | 0.00 | 0.01 |
|  | cg12030002 | 61237226 | 0.04 | 0.04 | 0.00 | 0.20 |
| **ESCA** | cg22215728 | 61236652 | 0.08 | 0.13 | 0.05 | 0.32 |
|  | cg15931943 | 61236909 | 0.07 | 0.07 | 0.00 | 0.82 |
|  | cg02854288 | 61236911 | 0.08 | 0.09 | 0.01 | 0.80 |
|  | cg19049316 | 61237063 | 0.03 | 0.03 | 0.00 | 0.80 |
|  | cg26322434 | 61237156 | 0.03 | 0.03 | 0.00 | 0.32 |
|  | cg24796403 | 61237172 | 0.03 | 0.03 | 0.00 | 0.60 |
|  | cg16986494 | 61237206 | 0.03 | 0.04 | 0.01 | 0.32 |
|  | cg12030002 | 61237226 | 0.03 | 0.03 | 0.00 | 0.32 |
| **KIRC** | cg22215728 | 61236652 | 0.12 | 0.08 | 0.04 | 0.00 |
|  | cg15931943 | 61236909 | 0.08 | 0.08 | 0.00 | 0.31 |
|  | cg02854288 | 61236911 | 0.08 | 0.08 | 0.00 | 0.70 |
|  | cg19049316 | 61237063 | 0.03 | 0.03 | 0.00 | 0.00 |
|  | cg26322434 | 61237156 | 0.03 | 0.03 | 0.00 | 0.69 |
|  | cg24796403 | 61237172 | 0.03 | 0.03 | 0.00 | 0.17 |
|  | cg16986494 | 61237206 | 0.03 | 0.03 | 0.00 | 0.00 |
|  | cg12030002 | 61237226 | 0.03 | 0.03 | 0.00 | 0.02 |
| **KIRP** | cg22215728 | 61236652 | 0.09 | 0.10 | 0.01 | 0.38 |
|  | cg15931943 | 61236909 | 0.08 | 0.08 | 0.00 | 0.73 |
|  | cg02854288 | 61236911 | 0.08 | 0.09 | 0.01 | 0.56 |
|  | cg19049316 | 61237063 | 0.03 | 0.03 | 0.00 | 0.98 |
|  | cg26322434 | 61237156 | 0.03 | 0.03 | 0.00 | 0.29 |
|  | cg24796403 | 61237172 | 0.04 | 0.03 | 0.01 | 0.56 |
|  | cg16986494 | 61237206 | 0.04 | 0.04 | 0.00 | 0.49 |
|  | cg12030002 | 61237226 | 0.04 | 0.04 | 0.00 | 0.75 |
| **LIHC** | cg22215728 | 61236652 | 0.13 | 0.15 | 0.02 | 0.35 |
|  | cg15931943 | 61236909 | 0.08 | 0.10 | 0.02 | 0.02 |
|  | cg02854288 | 61236911 | 0.09 | 0.10 | 0.01 | 0.09 |
|  | cg19049316 | 61237063 | 0.03 | 0.03 | 0.00 | 0.34 |
|  | cg26322434 | 61237156 | 0.03 | 0.03 | 0.00 | 0.04 |
|  | cg24796403 | 61237172 | 0.04 | 0.04 | 0.00 | 0.35 |
|  | cg16986494 | 61237206 | 0.04 | 0.05 | 0.01 | 0.02 |
|  | cg12030002 | 61237226 | 0.05 | 0.05 | 0.00 | 0.82 |
| **PRAD** | cg22215728 | 61236652 | 0.14 | 0.09 | 0.05 | 0.08 |
|  | cg15931943 | 61236909 | 0.08 | 0.08 | 0.00 | 0.20 |
|  | cg02854288 | 61236911 | 0.08 | 0.09 | 0.01 | 0.12 |
|  | cg19049316 | 61237063 | 0.03 | 0.03 | 0.00 | 0.94 |
|  | cg26322434 | 61237156 | 0.03 | 0.03 | 0.00 | 0.41 |
|  | cg24796403 | 61237172 | 0.04 | 0.04 | 0.00 | 0.82 |
|  | cg16986494 | 61237206 | 0.04 | 0.04 | 0.00 | 0.71 |
|  | cg12030002 | 61237226 | 0.04 | 0.05 | 0.00 | 0.51 |
| **THCA** | cg22215728 | 61236652 | 0.08 | 0.11 | 0.03 | 0.01 |
|  | cg15931943 | 61236909 | 0.09 | 0.09 | 0.00 | 0.75 |
|  | cg02854288 | 61236911 | 0.10 | 0.10 | 0.00 | 0.61 |
|  | cg19049316 | 61237063 | 0.03 | 0.03 | 0.00 | 0.61 |
|  | cg26322434 | 61237156 | 0.03 | 0.03 | 0.00 | 0.73 |
|  | cg24796403 | 61237172 | 0.04 | 0.04 | 0.00 | 0.84 |
|  | cg16986494 | 61237206 | 0.04 | 0.04 | 0.00 | 0.48 |
|  | cg12030002 | 61237226 | 0.04 | 0.04 | 0.00 | 0.64 |

McaM and McoM represent the mean of case methylation (Beta) and mean of control methylation (Beta). Methylation levels are calculated with formula: Beta = (M/M + U).

Position represents the chromosome position of each CpG site according to GRCh37/hg19.

P-values are derived from logistic regression analysis after FDR adjustment.
